# Genotypic Prediction of Co-receptor Tropism of HIV-1 Subtypes A and C

**DOI:** 10.1038/srep24883

**Published:** 2016-04-29

**Authors:** Mona Riemenschneider, Kieran Y. Cashin, Bettina Budeus, Saleta Sierra, Elham Shirvani-Dastgerdi, Saeed Bayanolhagh, Rolf Kaiser, Paul R. Gorry, Dominik Heider

**Affiliations:** 1 Department of Bioinformatics, Straubing Center of Science, University of Applied Sciences Weihenstephan-Triesdorf, Straubing, Germany; 2 Center for Biomedical Research, Burnet Institute, Melbourne, Australia; 3 Department of Bioinformatics, University of Duisburg-Essen, Essen, Germany; 4 Institute of Virology, University of Cologne, Cologne, Germany; 5 Viral Hepatitis and Immunobiology Lab, University Hospital Aachen, Aachen, Germany; 6 Iranian Research Center of HIV /AIDS, Tehran University of Medical Sciences, Tehran, Iran; 7 School of Applied Sciences, and Program in Metabolism, Exercise and Disease, Health Initiatives Research Institute, RMIT University, Melbourne, Australia; 8 Wissenschaftszentrum Weihenstephan, Technische Universität München, Freising, Germany

## Abstract

Antiretroviral treatment of Human Immunodeficiency Virus type-1 (HIV-1) infections with CCR5-antagonists requires the co-receptor usage prediction of viral strains. Currently available tools are mostly designed based on subtype B strains and thus are in general not applicable to non-B subtypes. However, HIV-1 infections caused by subtype B only account for approximately 11% of infections worldwide. We evaluated the performance of several sequence-based algorithms for co-receptor usage prediction employed on subtype A V3 sequences including circulating recombinant forms (CRFs) and subtype C strains. We further analysed sequence profiles of gp120 regions of subtype A, B and C to explore functional relationships to entry phenotypes. Our analyses clearly demonstrate that state-of-the-art algorithms are not useful for predicting co-receptor tropism of subtype A and its CRFs. Sequence profile analysis of gp120 revealed molecular variability in subtype A viruses. Especially, the V2 loop region could be associated with co-receptor tropism, which might indicate a unique pattern that determines co-receptor tropism in subtype A strains compared to subtype B and C strains. Thus, our study demonstrates that there is a need for the development of novel algorithms facilitating tropism prediction of HIV-1 subtype A to improve effective antiretroviral treatment in patients.

Human immunodeficiency virus type 1 (HIV-1) requires multiple steps to gain entry into CD4-expressing cells. The initial step comprises the binding of the gp120 subunit of the envelope protein complex (env) to the cell surface receptor CD4 of host cell membranes. For viral entry, the viral gp120 also needs to bind to one of the two secondary cell-surface co-receptors, namely CXCR4 or CCR5, to activate fusion of the virus and the host cell^1^. Depending on the usage of CXCR4 or CCR5 the isolates (viral samples) are called X4 or R5, respectively. Viruses with tropism for both co-receptors are called ‘dual-tropic’ or R5X4. The third hypervariable region (V3 loop) in the gp120 protein has been recognised as the major determinant for co-receptor tropism of the isolates^2^. However, further regions of HIV-1 gp120 outside of the V3 loop have also been linked to co-receptor tropism^3^,^4^.

The development of the entry inhibitor Maraviroc has made it feasible to prevent viral entry through specific binding to the CCR5 co-receptors on the host cells and therefore inhibiting further CCR5-virus interactions^5^. However, this drug is only effective in suppressing viral replication in patients harbouring R5 populations and is contraindicated in patients with circulating X4 and R5X4 viruses. Unfortunately, HIV-1 strains are able to switch their co-receptor usage: while patients at early infection stages harbour only R5 viruses as predominant isolates, in advanced stages of the disease X4 and R5X4 viruses emerge in approximately 50% of patients infected with subtype B viruses^6^,^7^. Thus, it is crucial to precisely predict co-receptor usage in patients before administering CCR5-blocking drugs^8^ to achieve an effective antiretroviral treatment. So far, two types of methodologies have been developed for assessing viral tropism: (i) *in vitro* phenotypic tests, which are cell-based, such as *Trofile^®^ (Monogram Biosciences)*, and (ii) *in silico* methods that are based on viral genotypic information. Although phenotypic tests of co-receptor tropism have a high sensitivity, they require specialised laboratories, are expensive and have a long turn-around time (2–3 weeks)^9^. Due to computational advances, *in silico* prediction methods have become relatively low-cost and more rapid alternatives.

Most of the genotypic prediction models are based on sequence information of the viral V3 loop derived from plasma samples of the patients. One of the first tropism prediction approaches was the 11/25 rule, which asserts a virus as X4-tropic if amino acids at either positions 11 and 25 of the V3 loop, which is around 35 amino acids in length, are positively charged^10^. In recent years, more sophisticated models have been developed that outperform the 11/25 rule, e.g. support vector machines^11^, artificial neural networks^12^, structural models^13^ and position specific scoring matrices (PSSM)^14^. The most commonly used tools today are geno2pheno^15^ and WebPSSM^14^, which deliver high levels of sensitivity^16^. Nevertheless, the performance of computational models in tropism prediction of HIV-1 strongly depends on the database that has been used for algorithm development. Most of the models have been trained on V3 sequences derived from subtype B strains, thus it is questionable whether these models are reliable for predictions of non-B strains.

HIV can be separated into two species, namely HIV-1 and HIV-2. Both species are independently transferred from different primates to humans, whereas HIV-1 infections constitute around 90% of all infections worldwide^17^. In contrast to HIV-1, HIV-2 is less infective and less virulent. HIV-1 strains can further be separated into three major subgroups: M (main), O (Outlier, consisting of a small number of viruses from Cameroon) and N (non-M, non-O)^17^. The HIV-1 subtypes are defined based on their genetic diversity and on specific mutation patterns and recombinations^18^. The highest diversity can be found in the envelope protein, in particular within the glycoprotein 120 (gp120). The *env* gene encodes for the surface glycoprotein 120 and the transmembrane glycoprotein 41 (gp41), which are involved in viral entering of host cells and in co-receptor usage^19^,^20^.

Overall, M is the major subgroup of HIV-1 comprising around 97% of HIV-1 infections, and further divided into subtypes: A, B, C, D, F, G, H, J, K and circulating recombinant forms (CRFs). CRFs represent recombination of distinct subtypes, for instance CRF02_AG describes a recombination between subtype A and G. The world’s most prevalent subtype is subtype C, accounting for around 48% of all HIV-1 infections, and is predominantly distributed in Sub-Saharan Africa and Central Asia. Subtype A and its CRFs constitute the second most prevalent group of around 25% and predominate in countries such as Tanzania, Kenya, Angola, Chad, Madagascar, Kazakhstan, Iran and Russia. Only around 11% of HIV-1 infections are caused by subtype B, which mainly spreads in developed regions such as Europe and North America.

In the current study, we evaluated the performances of state-of-the-art methods for tropism prediction of sequences derived from subtype A and C strains, namely T-CUP 2.0^21^, geno2pheno_[coreceptor]_^15^, PhenoSeq^22^, WebPSSM^14^ using all matrices, i.e. x4r5, sinsi and sinsi c, and the genotypic rules of Raymond *et al.*^9^ and Esbjörnsson *et al.*^23^, respectively. An overview of the applied algorithms is provided in Table 1. Furthermore, we analysed differences of sequence profiles of gp120 sequences from subtype A, B and C to discover functional relationships to entry phenotypes. This might offer new targets capable of inferring improved co-receptor usage prediction especially for subtype A.

## Results and Discussion

In the current study, we evaluated the performances of V3 loop-based algorithms on non-B strains, i.e. subtype C and A including CRFs. As we found that current tools are not applicable to predict subtype A tropism, we subsequently checked whether different molecular mechanisms contribute to the co-receptor determination in subtype A compared to subtype B and C strains. By means of profiling subtype A gp120 sequences, we detected significant associations outside the V3 region that could contribute to co-receptor tropism in subtype A strains. The V2 region in the subtype A sequence profile shows a statistically higher association to tropism than the V3 loop in contrast to subtype B and C strains. Furthermore, the development of random forest models solely trained on subtype A sequences, which generally work well on subtype B sequences, are not able to separate R5 and X4 isolates. Thus, there is a strong indication that further mechanisms outside the V3 loop determine co-receptor tropism in subtype A strains and CRFs.

Overall, we used a dataset consisting of 56 sequences derived from X4-using viruses and 359 sequences from R5-tropic subtype C viruses. For subtype A 209X4 and 190 R5 sequences were used. Co-receptor usage prediction was then conducted using three computational approaches with different design of algorithms: T-CUP 2.0, geno2pheno and PhenoSeq. Additionally, we applied the scoring matrices x4r5, sinsi and sinsi for subtype C of WebPSSM and the genotypic rules according to Raymond *et al.* and Esbjörnsson *et al.*

### Comparison of prediction performances

Table 2 shows performance results of the different algorithms for subtype A and C. For subtype C, all methods achieved a specificity of more than 92%, with the rule of Raymond *et al.* showing the best performance (99.4%). Highest sensitivity was attained by PhenoSeq (91.38%). The accuracy of all approaches ranged from 91.17% to 98.09%, whereas the rule of Raymond *et al.* demonstrated the best overall performance.

For subtype A, high specificity values were obtained with all methods ranging from 93.94% (WebPSSM x4r5) to 99.49% (Esbjörnsson), except for WebPSSM sinsi C where the specificity was much lower (58.59%). However, sensitivity was heavily decreased in all methods compared to subtype C, with an overall performance resulting in sensitivity values lower than 20%. The highest sensitivity was achieved by WebPSSM sinsi C with 37.8%, nevertheless the specificity (58.59%) and accuracy (47.81%) were lower compared to the other approaches where the specificity and accuracy range from 93.94% to 99.49% and 53.56% to 55.39%, respectively. Notably, the recently developed PhenoSeq tool exhibited limited sensitivity (17.7%), specificity (94.74%) and accuracy (54.39%) for subtype A predictions, despite comprising a subtype A specific tropism test. As discussed here, and by the PhenoSeq authors^22^, additional sequence information within gp120 and/or gp41 is likely required to improve the performance of genotypic tropism tests that are specific for subtype A.

Albeit having accurate prediction performance in determining the tropism of HIV-1 subtype C, tropism prediction of co-receptor usage for subtype A sequences resulted in low accuracies by all methods (~54%). Remarkably, while R5-tropic subtype A viruses could be detected at a high rate, the detection of X4 or dual-tropic isolates was less accurate. The DOR was found to be lower in subtype A predictions compared to subtype C predictions for all approaches.

**Figure 1:**
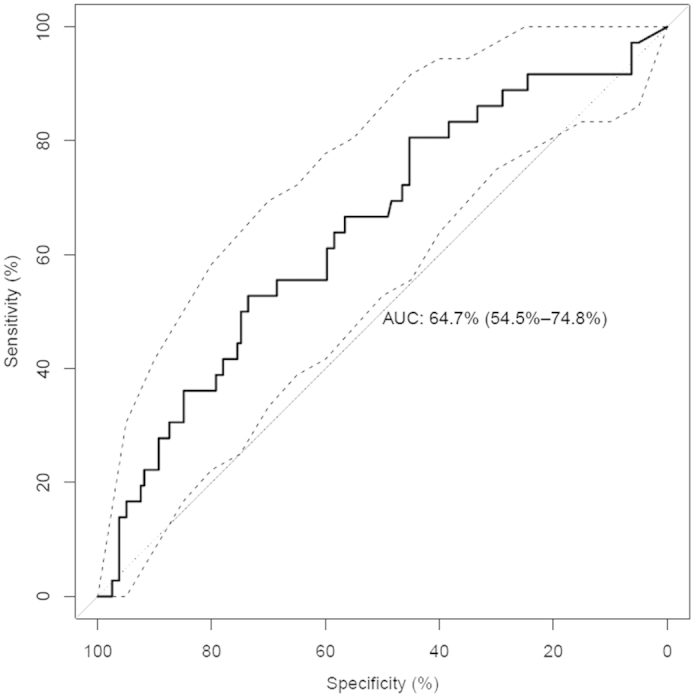
ROC-Curve of the best performing descriptor. The ROC-Curve with confidence intervals of the best performing descriptor on subtype A V3 sequences is shown. A random forest model was used to classify sequences as X4 vs. R5. The sequences were encoded with the Zimm-Brag parameter sigma.

Due to the low prediction accuracy for subtype A sequences, we tested whether the available tools show low predictive capacity because of missing training data or inappropriate design for subtype A strains. Therefore, we constructed random forest models to predict co-receptor usage of subtype A viruses incorporating 159 R5 sequences and 36X4 sequences of V3 loop region. This dataset is a subset of the subtype A dataset (see Methods), with only one sequence per patient. For data representation, amino acids were encoded using hydrophobicity scores according to Kyte and Doolittle^24^, as this descriptor has been shown to achieve good predictive performance in former classification tasks, e.g. as applied in T-CUP 2.0 on subtype B sequences. The resulting model achieved an AUC of 0.5765 +/− 0.0084, which is only slightly better than random guessing as assessed via permutation tests. Therefore, we tested whether different numerical representations would increase prediction performance for subtype A and applied all available descriptors from the AAindex database for encoding of sequences. The best working descriptor (Zimm-Brag parameter sigma^25^) achieved an AUC of 0.6467 +/− 0.0092, however the AUC was not significantly higher compared to the one obtained from the hydrophobicity descriptor (p = 0.09768). Figure 1 shows the ROC curve for the Zimm-Brag parameter sigma representation. However, the overall performance was still not comparable to the performance of the other models on subtype B and subtype C. Thus, we used a statistical analysis to identify features in gp120 that might have relevant impact on molecular mechanisms accounting for co-receptor tropism within the different subtypes.

### Sequence profiling of gp120 sequences of strains A, B and C

**Figure 2:**
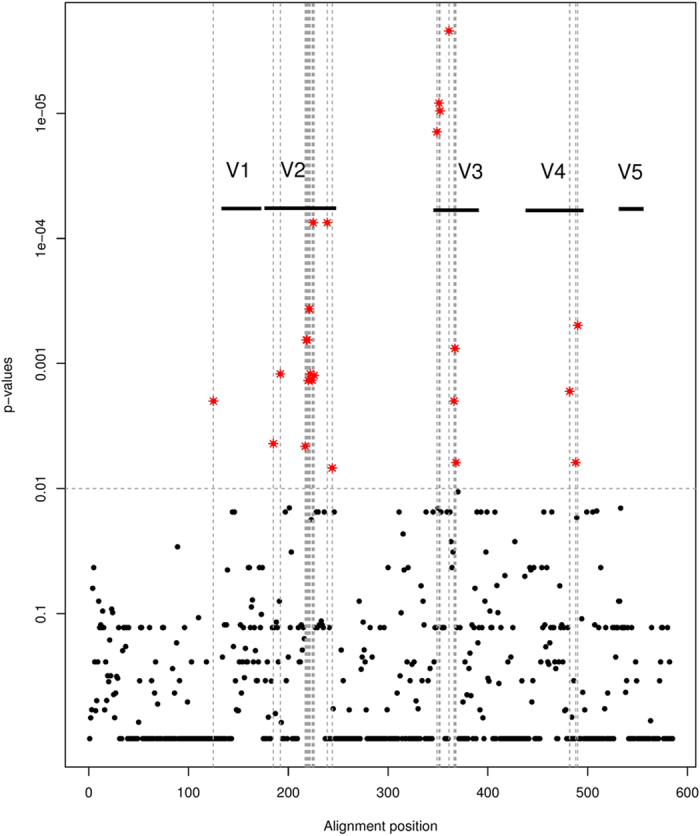
Association plot of subtype A. On the x-axis alignment positions of gp120 region are shown, the y-axis represents the associated p-values based on SeqFeatR. Significant changes in amino acid composition between X4 and R5 sequences (p-value < 0.01) are marked with asterisks. The variable regions V1–V5 of gp120 are drawn. The strongest associations were found in the V3 and V2 regions. Additionally, the region of V4 shows statistically significant associations to co-receptor tropism.

**Figure 3:**
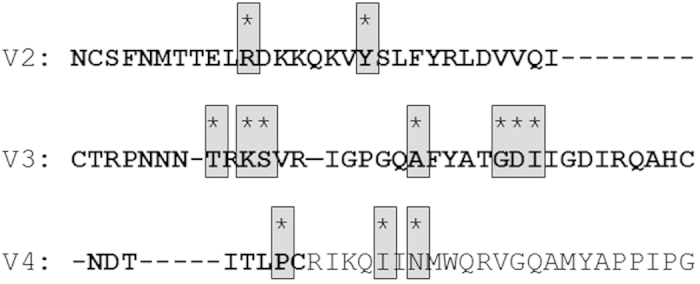
Significant changes in subtype A sequences. Consensus sequences of V2, V3 and V4 are shown for subtype A. Significant changes of amino acid compositions that have been detected with SeqFeatR are highlighted in grey and marked with asterisks. The loops are shown in bold. The regions around V3 and V2 show strongest statistical differences in amino acid composition.

Sequence logos of X4 and R5 subtype A sequences were calculated and are shown in supplementary Figures 1 and 2. General differences in amino acid composition were observed predominantly at five positions: 5, 13, 15, 20 and 26. Sequence logos for subtype B and subtype C sequences are shown in supplementary Figures 3–6. Moreover, we analysed which amino acid alterations in the sequences of gp120 (consisting of variable regions V1–V5 and constant segments C1–C5) of subtype A might have an impact on co-receptor tropism. Indeed, the importance of the V1/V2 region in the specificity of co-receptor usage has been documented already^26^,^27^,^28^. In the current study, we performed a multiple sequence alignment of subtype A-derived gp120 sequences to discover associations of amino acids with tropism at each alignment position using SeqFeatR. We observed five significantly different regions within gp120. The association plot is shown in Fig. 2. The most significant differences in amino acid composition could be observed for positions located in the V3 region, confirming V3 as the major tropism determinant. However, three regions outside V3 were detected with significant differences in sequence composition between X4- and R5-using strains, including the hypervariable region V2 (p < 0.0001), a region located close to V2, and a third region neighbouring V4 (see Fig. 3).

**Figure 4:**
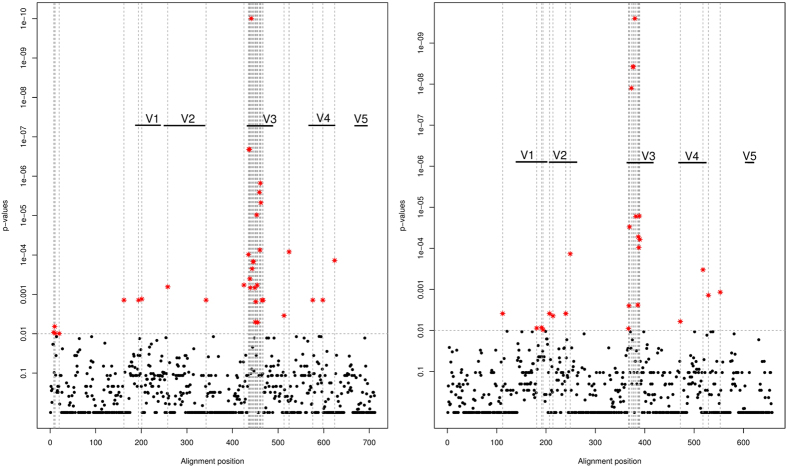
Association plot of subtype B and subtype C. On the x-axis alignment positions of gp120 region are shown for subtype B (left) and C (right), the y-axis represents the associated p-values based on SeqFeatR. Significant differences in amino acid composition between X4 and R5 sequences can be observed at V3 region in both subtypes with the strongest signals. In addition, regions around V1, V2 and V5 show significant differences.

In order to compare these findings with subtype B and C, we also calculated multiple sequence alignments for gp120 sequences derived from subtype B and C and subsequently used the SeqfeatR package again to discover significant associations of amino acid composition. The association plots are shown in Fig. 4. Overall, the highest significant association was observed for both subtypes in the region of V3 (positions in the subtype C alignment at around 390, subtype B positions at around 440). The statistical significance in subtype B (p < 1.0 × 10^−10^) and C (p < 1.0 × 10^−9^) for the V3 region was much higher compared to subtype A (p < 1.0 × 10^−6^). Interestingly, significant associations were identified around the V2 region in both subtypes; however, the strongest association signal was detected for the V2 region in subtype A. For subtype C a significant association was observed within the CD4-binding-loop.

Substitutions in gp120 regions outside the V3 loop and in gp41 have been shown to influence co-receptor usage^4^,^29^,^30^. It is known that V2 is a component of the co-receptor-binding site and mutations in this region may mediate R5-to-X4 switch in certain cases^26^. In addition, it has been shown that mutations in V3 are typically associated with tropism switch leading to viral lethality or decrease in viral fitness, unless they are compensated by mutations in the V1/V2 region^28^. The mechanism of this compensation is unknown but may be of a structural nature as the V1/V2 region of gp120 is thought to be located close to the trimer interface^31^. V2 loops physically interact with the V3 loops in the trimeric form of the Env spike to constitute 3D-conserved motifs. This 3D conservation assures the functionality of the Env spike and allows motif recognition by different cross-reacting antibodies^32^. There are no data regarding either the prevalence or the fitness effects of V1/V2 compensatory mutations for any HIV-1 subtype, though this may be an explanation for the different frequency of tropism among subtypes, ranging from 30% for subtype C to 70–80% in subtype A^23^.

## Conclusions

In our study, we demonstrated that currently available approaches for co-receptor usage prediction are not capable of determining co-receptor tropism of subtype A sequences. Admittedly, prediction algorithms work well on subtype B, on which they were developed, and also showed high prediction accuracy for subtype C. However, on subtype A and CRFs, the currently existing algorithms displayed low prediction performances with sensitivity less than 20%. Furthermore, we demonstrated that X4 and R5 viruses of subtype A could not be distinguished reliably by means of predictive models trained specifically on subtype A sequences.

By analysing sequence composition of near full-length gp120 of subtype A, we found significant differences between X4 and R5 strains inside and outside the V3 region. This is in line with published findings, e.g. by Thielen *et al.*^33^, indicating the improving role of V2 domain incorporation in the function of tropism prediction algorithms and refers to further molecular mechanisms which are involved in co-receptor usage. However, this was only shown for subtype B so far. We have not been able to build predictive models for co-receptor tropism based on complete gp120 sequences due to the low number of available sequences with tropism information. Future approaches should include reliable phenotypic tropism determination and gp120 sequencing to permit the elaboration of models to explore the possible benefit of including additional gp120 regions in the tropism determination of non-B samples.

Overall, our study showed that there is a need for the development of novel algorithms facilitating tropism prediction of HIV-1 subtype A to improve effective antiretroviral treatment for HIV-1 infected patients, especially in low and middle income countries where such strains predominate. Further preprocessing of the sequence data, e.g. by using feature extraction methods^34^ could be used to improve prediction performance. Additional information, such as sequence-derived information in combination with structural information^35^,^36^ of the V3 loop could also increase accuracy.

## Methods

### Data

V3 loop sequences of HIV-1 with assigned subtypes C, A or CRFs were retrieved from the Los Alamos HIV sequence database (http://hiv-web.lanl.gov/) in March 2015. Sequences with ambiguities were removed. Additionally, we used nine subtype A and three subtype C V3 sequences that were collected at the Institute of Virology at the University of Cologne. Overall, 56X4-using viruses (28X4 and 28 R5X4), and 359 R5 viruses were used for subtype C analyses. We included a total of 190 R5 and 209X4-using viruses for subtype A (and CRFs) as follows: *i)* three X4 and 40 R5 sequences classified as subtype A, *ii)* five X4, five R5X4 and 58 R5 subtype A1 samples; *iii)* 11X4, 185 R5X4 and 88 R5 CRF02_AG samples; *iv)* one R5 of CRF11_cpx; and *v)* three R5 of CRF22_01A1. All of the CRFs have a V3 region originating from subtype A. Moreover, we collected full length gp120 sequences from subtype A (74 CCR5 and 11 CXCR4 sequences), B (254 CCR5 and 34 CXCR4 sequences) and C (168 CCR5 and 22 CXCR4 sequences) from the Los Alamos HIV sequence database.

### Phylogenetic analysis of the samples

To confirm the Los Alamos-assigned subtypes of our sequences, we performed a phylogenetic analysis. The multiple sequence alignment of V3 sequences was computed with MUSCLE^37^. The trees were calculated with SeaView 4^38^ using Poisson distance and BioNJ, a distance based phylogeny tree-building algorithm. Gap sites were ignored and significance was estimated by bootstrapping with 100 replicates.

### Genotypic prediction

For co-receptor usage prediction we compared the performance of the following tools: T-CUP 2.0^21^, geno2pheno_[coreceptor]_^15^, PhenoSeq^22^ and WebPSSM^14^. For geno2pheno_[coreceptor]_ we used a false positive rate (FPR) cutoff of 5%, whereas T-CUP 2.0 and PhenoSeq were used with standard settings. WebPSSM was used with all available matrices: x4r5, sinsi and sinsi for subtype C. We further employed the genotypic rules of Raymond *et al.*^9^, as well as one rule proposed by Esbjörnsson *et al.*^23^ for subtype A with a cut-off of 5 for the mean net charge and a cut-off of 8 for the total count of charged amino acids.

### Construction of random forest models

Besides the application of existing approaches, we used the randomForest package^39^ in R to develop new classification models specifically for co-receptor tropism prediction of subtype A sequences. For data representation amino acid positions of V3 loop sequences were encoded with descriptors of the AAindex database^40^ and subsequently interpolated to a uniform length of 35 using the Interpol package^41^. Random forests were trained with a leave-one-patient-out cross-validation scheme repeated 10 times. Performances were measured by calculating the area under the receiver operating characteristic (ROC) curve (AUC).

### Performance measures

For the assessment of prediction performance of all prediction algorithms, we used common measures for classification tasks, whereas TP denotes the count of true positives, TN true negatives, FP false positives and FN false negatives. The measures are defined as following:


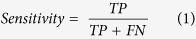



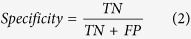



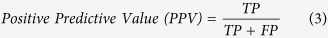



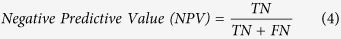



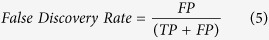



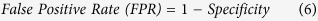



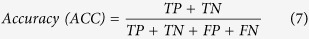



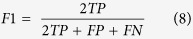


Further, we calculated the diagnostic odds ratio (DOR)^42^, which is a measure of the effectiveness of a diagnostic test. In our case, the DOR of a model is defined as the ratio of the odds of a positive prediction (i.e. X4) in case of a looking at an X4 isolate compared to the odds of a positive prediction in case of a R5 isolate:


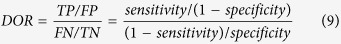


The DOR ranges from zero to infinity. Higher values indicate better test performances. As the DOR is not defined in cases where the denominator is 0, we added 1 to all values, and additionally, rounded the DOR to integers. Moreover, we performed permutation tests to evaluate the robustness of our models^43^,^44^.

### Sequence analysis of gp120

To analyse which amino acid alterations in the sequences of gp120 of subtype A may have an impact on co-receptor tropism, we downloaded all available subtype A gp120 sequences with tropism information from the Los Alamos HIV Database (74 CCR5 and 11 CXCR4 sequences), calculated a multiple sequence alignment using MUSCLE, and subsequently used the R-package SeqFeatR^45^ to discover associations of amino acids with tropism at each alignment position. SeqFeatR constructs 2 × 2 contingency tables with counts of the occurring combinations of tropism and amino acid for each position, and then executes Fisher’s exact tests to discover significant associations. We did the same for gp120 sequences derived from subtype B (254 CCR5 and 34 CXCR4 sequences) and subtype C (168 CCR5 and 22 CXCR4 sequences). Sequence logos of V3 loop regions were created using WebLogo 3^46^.

## Additional Information

**How to cite this article**: Riemenschneider, M. *et al.* Genotypic Prediction of Co-receptor Tropism of HIV-1 Subtypes A and C. *Sci. Rep.* **6**, 24883; doi: 10.1038/srep24883 (2016).

## Supplementary Material

Supplementary Information

## Figures and Tables

**Table 1:** Overview of computational tools.

Tool	Prediction method	Subtype
T-CUP 2.0	Uses structural information of the V3 loop by modelling the electrostatic potential and hydrophobicity; combination of results by stacking	subtype B (primarily) and C with 1351 sequences (200X4 and 1151 R5)
Geno2pheno_[coreceptor]_	Support vector machine for binary classification	subtype B with 1100 sequences (769 R5, 210X4, 131 R5X4) from 332 patients
PhenoSeq	Evaluation of HIV-1 V3 amino acid length, net amino acid charge, number of N-linked glycosylation sites and the frequency of site-specific amino acid alterations	A, A1, A2, B, C, D, CRF_01_AE, CRF02_AG
WebPSSM x4r5	Scoring matrices, reflecting the difference in abundance of a particular amino acid at a particular site	subtype B
WebPSSM sinsi	Scoring matrices, reflecting the difference in abundance of a particular amino acid at a particular site	subtype B
WebPSSM sinsiC	Scoring matrices, reflecting the difference in abundance of a particular amino acid at a particular site	subtype C
Genotypic rule (Raymond *et al.*)	11/25 rule in combination with a net charge rule	subtype C
Genotypic rule (Esbjörnsson *et al.*)	Rules based on Raymond *et al.* with modified cut-offs of the mean net charge and total count of charged amino acids	subtype A

**Table 2:** Prediction performance of co-receptor usage models.

Subtype	Method	Sens	Spec	Acc	PPV	NPV	FPR	FDR	F1	DOR
**C**	T-CUP	91.07	98.60	97.59	91.07	98.61	1.39	8.93	91.07	513
	g2p	87.50	97.77	96.39	85.96	98.04	2.23	14.04	86.73	244
	PhenoSeq	91.38	92.48	92.33	75.71	98.56	4.74	24.29	82.81	172
	X4R5	75.00	94.49	91.89	67.74	96.08	5.51	32.26	71.19	47
	SINSI	71.43	98.90	95.23	90.91	95.73	1.10	9.09	80.00	174
	SINSI.C	89.29	91.46	91.17	61.73	98.22	8.54	38.27	72.99	76
	Raymond	89.29	99.45	98.09	96.15	98.37	0.55	3.85	92.59	879
**A**	T-CUP	18.18	96.32	55.39	84.44	51.69	3.68	15.56	29.92	5
	g2p	15.79	97.89	54.89	89.19	51.38	2.11	10.81	26.83	7
	Phenoseq	17.70	94.74	54.39	78.72	51.14	5.26	21.28	28.91	4
	X4R5	15.31	93.94	53.56	72.73	51.24	6.06	27.27	25.30	3
	SINSI	11.54	97.98	53.69	85.71	51.32	2.02	14.29	20.34	5
	SINSI.C	37.80	58.59	47.91	49.07	47.15	41.41	50.93	42.70	1
	Raymond	11.00	98.48	53.56	88.46	51.18	1.52	11.54	19.57	6
	Esbjörnsson	13.40	99.49	55.28	96.55	52.12	0.51	3.45	23.53	16

For subtype C each algorithm achieved a sensitivity of around 90%. Prediction performance for subtype A generally resulted in sensitivities lower than 20%.
