# Supplementary material for: Genotypic Prediction of Co-receptor Tropism of HIV-1 Subtypes A and C
Source: Sci Rep. 2016 Apr 29;6:24883. doi: 10.1038/srep24883 (PMC4850382; doi:10.1038/srep24883)
Supplement: Supplementary Information [file srep24883-s1.pdf]

SUPPLEMENT

Genotypic Prediction of Co-receptor Tropism of HIV-1 Subtypes A and C

Mona Riemenschneider, Kieran Y. Cashin, Bettina Budeus, Saleta Sierra, Elham Shirvani-Dastgerdi, Saeed Bayanolhagh, Rolf Kaiser, Paul R. Gorry, Dominik Heider

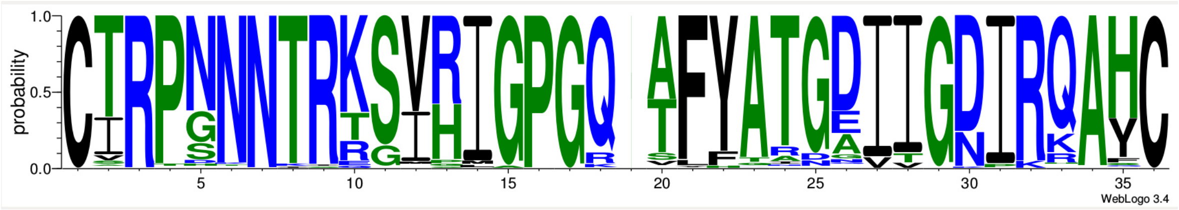

Figure S1: Logo of subtype A R5 sequences.

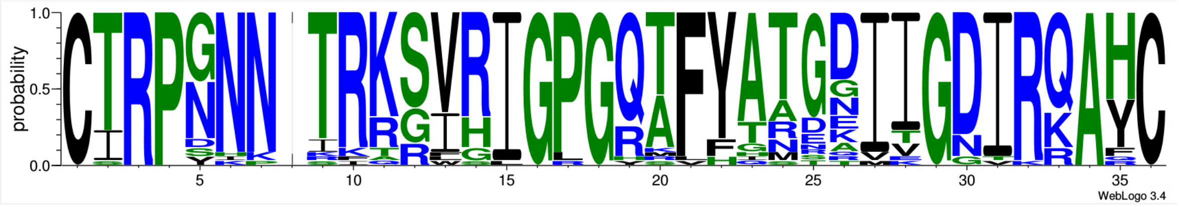

Figure S2: Logo of subtype A X4 sequences.

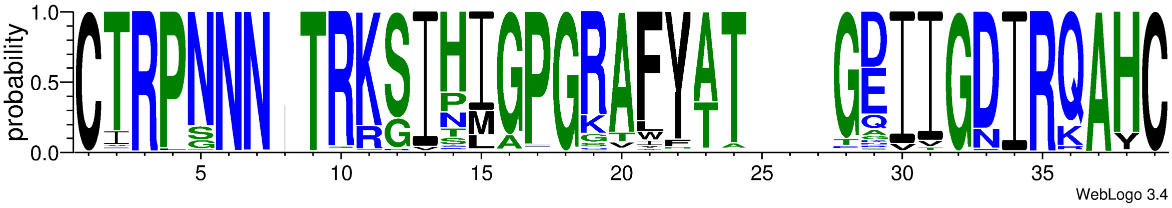

Figure S3: Logo of subtype B R5 sequences.

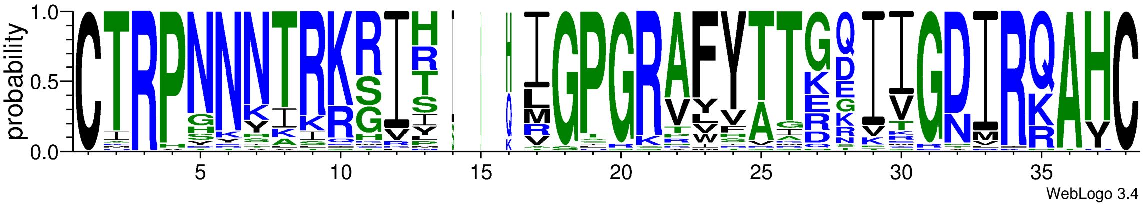

Figure S4: Logo of subtype B X4 sequences.

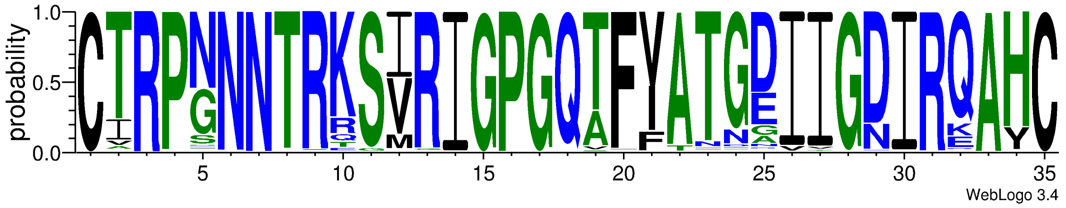

Figure S5: Logo of subtype C R5 sequences.

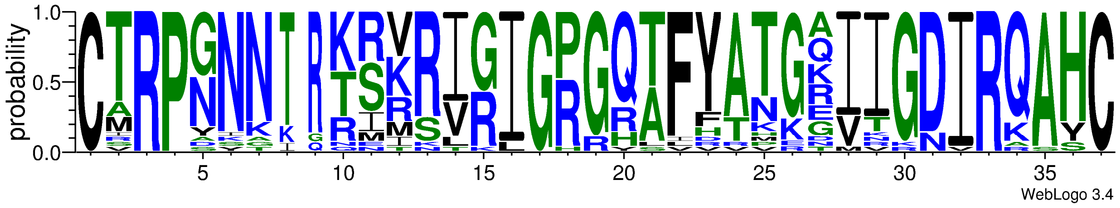

Figure S6: Logo of subtype C X4 sequences.
